# The Humanized Mouse Model: What Added Value Does It Offer for HIV Research?

**DOI:** 10.3390/pathogens12040608

**Published:** 2023-04-17

**Authors:** Luca Baroncini, Simon Bredl, Kadzioch P. Nicole, Roberto F. Speck

**Affiliations:** Department of Infectious Diseases and Hospital Epidemiology, University Hospital of Zurich, University of Zurich, 8091 Zurich, Switzerland

**Keywords:** hu mice, HIV, human hematopoietic stem and progenitor cells, bNAbs, gene therapy, pathogenesis

## Abstract

In the early 2000s, novel humanized mouse models based on the transplantation of human hematopoietic stem and progenitor cells (HSPCs) into immunocompromised mice were introduced (hu mice). The human HSPCs gave rise to a lymphoid system of human origin. The HIV research community has greatly benefitted from these hu mice. Since human immunodeficiency virus (HIV) type 1 infection results in a high-titer disseminated HIV infection, hu mice have been of great value for all types of HIV research from pathogenesis to novel therapies. Since the first description of this new generation of hu mice, great efforts have been expended to improve humanization by creating other immunodeficient mouse models or supplementing mice with human transgenes to improve human engraftment. Many labs have their own customized hu mouse models, making comparisons quite difficult. Here, we discuss the different hu mouse models in the context of specific research questions in order to define which characteristics should be considered when determining which hu mouse model is appropriate for the question posed. We strongly believe that researchers must first define their research question and then determine whether a hu mouse model exists, allowing the research question to be studied.

## 1. Introduction

Human immunodeficiency virus (HIV) is a human pathogen, categorized into HIV type 1 and type 2. HIV type 1 is the virus that caused the global pandemic, while HIV type 2 is mostly confined to West Africa [1]. This review focuses on HIV type 1, unless otherwise noted. Thus, “HIV” in this review text refers to HIV-1.

Conventional small rodents used in biomedical research are resistant to HIV. Alternatives are non-human primates that are susceptible to the closely related simian immunodeficiency virus (SIV), which have been highly valuable for HIV research [2]. However, for ethical and cost reasons, work with non-human primates is limited to very few laboratories worldwide. Furthermore, data from SIV-infected non-human primates cannot be directly extrapolated to humans infected with HIV. Humanized mice were used to study HIV infection as early as the 1980s. The earliest models were the SCID-hu Thy/Liv mouse model and the hu-PBL-SCID mouse model [3] (see Box 1 for the nomenclature of humanized mice). Both models, while valuable for HIV research at the time, have significant limitations, including restriction of the analysis to the human artificial lymphoid organ under the renal capsule in the case of the SCID-hu Thy/Liv mouse model, as well as graft versus host disease (GvHD) in the case of the hu-PBL-SCID model. A new chapter in humanized mouse research began with the injection of human hematopoietic stem and progenitor cells (HSPCs) into severely immunocompromised mice. The human HSPCs engraft within three to four months and result in a hemato-lymphatic system of human origin [4]. All types of human immune cells are found in these humanized mice, which may differ in their quantity or quality according to the mouse background used. Unless otherwise indicated, hu denotes mice, e.g., hu NSG, hu BRG, humanized by transplantation of HSPCs by any method. When we use the word “humanized”, we would be referring to mice, which have been reconstituted with any type of immune cells or lymphoid tissue, including hu mice and bone marrow (B)–liver–thymus (BLT) mice. Here, we first outline the core questions of HIV research and then discuss the various humanized mouse models and to what extent they are useful in investigating the various research questions.

The review does not offer a structured overview of the existing humanized mouse models with their advantages or disadvantages or a comparison of different humanized mouse models independent of HIV; we refer the reader to recently published reviews on this topic [4,5,6,7]. Furthermore, we do not cover the work on the original hu-PBL-SCID and SCID-hu Thy/Liv mice.

Box 1 **Nomenclature of humanized mice.**

BRG: *Rag2−/−/IL-2Rγ−/−* in BALB/c background

BRGS: Balb/c *Rag2−/−IL-2Rγc−/−Sirp-α^NOD^* (BRG mice congenic for the NOD.sirp-α gene, which interacts with human CD47)

DRAG: Human HLA-DR4 molecule in NRG

DRAG-A2 or DRAGA: Human HLA-DR4 and HLA-A2 molecules in NRG

MISRTG: *M-CSF*, *IL-3/GM-CSF*, *SIRP-α*, TPO knock-in in *RAG2−/−IL-2Rγ−/−* background

MITRG: *M-CSF*, *IL-3/GM-CSF*, *TPO* knock-in in RAG2−/−IL-2Rγ−/− background

NRG: *Rag1−/−/IL-2Rγ−/−* in NOD background

NSG/NOG: SCID/*IL-2Rγ−/−* in NOD background; the NSG mouse has a complete null allele for the IL-2Rγ chain while the NOG mouse lacks IL-2Rγ intracytoplasmic domain (cytokine binding but no signaling)

NSG-SGM3: Human Stem cell factor, *GM-CSF*, *IL-3* in NSG background

SCID: Genetic autosomal recessive mutation in the Prkdc*^scid^* gene

SRG-15: Human *SIRP-α* and human *IL-15* knock-in in NSG background

TKO: *Rag2−/−γc−/−CD47−/−* in C57BL/6 background

## 2. What Are the Major Questions in HIV Research?

What is the major question in HIV research? We simplify it as follows: how can HIV infection be cured? Combined anti-retroviral treatment (cART) has dramatically changed the prognosis of patients living with HIV; however, it is unable to cure HIV. The widespread use of cART has also revealed its disadvantages—among others, adverse events, the emergence of drug-resistant strains, immune activation despite cART, the need for daily intake and, thus, treatment fatigue, and social stigma. In addition, UNAIDS is working towards the 90-90-90 goal (90-90-90: treatment for all | UNAIDS), i.e., treatment for all in order to end the HIV pandemic; however, we are still far from this ambitious target and this work is currently made more difficult by the COVID-19 pandemic and military conflicts.

Research questions are always subject to investigators’ preferences. Here, we list our identified challenges, as related to novel treatment approaches.

Testing of novel anti-HIV compounds and anti-HIV broadly neutralizing antibodies (bNAbs): Today, HIV doctors possess a fairly large arsenal of conventional drugs to treat HIV-infected patients [8]. Fortunately, the search for even better anti-HIV drugs continues. In particular, compounds are being investigated that target key HIV replication steps other than the conventional replication steps, e.g., compounds deactivating provirus transcription (“block-and-lock”) [9] and those that have fewer side effects or that have very long pharmacokinetic half-lives.

Furthermore, bNAbs, bi-specific antibodies, and antibody mimetics such as DARPins [10], have come to the fore in the past decade in the fight against the HIV pandemic. All of these novel agents must be tested individually or in combination, preferentially in a small-animal model, in order to obtain a pre-clinical proof of concept.

Development of cell and gene therapies to cure HIV: HIV depends on critical host factors for the completion of its replication cycle. Together with the cure of HIV infection in single patients following stem cell transplants from donors lacking CCR5 (CCR5 Δ32 homozygous donors) [11], this fact underscores the potential of genetic engineering to cure HIV. One promising approach is the genetic removal of CCR5 from hematopoietic stem and progenitor cells (HSPCs), rendering the progeny cells non-permissive to CCR5-tropic HIV strains [11]. Other approaches are based on genetically engineering either B cells for the expression of bNAbs or HIV-specific T cells resistant to HIV infection [12]. Essentially, all current clinical trials of gene therapies are based on the adoptive, either autologous or allogeneic, cell transfer of genetically engineered cells [11]. Therefore, the greatest challenge is to identify vectors for in vivo gene therapy that obviate the need for chemotherapeutic pre-conditioning [13], which is needed to provide space in adoptive cell therapies.

Exploration of experimental strategies to eradicate dormant HIV: The introduction of cART was one of the major breakthroughs in HIV medicine, resulting in a dramatic decline in morbidity and mortality among HIV-infected patients [14]. However, cART is not able to cure HIV because cART solely targets active HIV replication steps; HIV is able to hibernate in various immune cells in a latent state [15]. Various approaches have been explored to target these latently HIV-infected cells. The imminent barrier to doing so is the lack of any unequivocal marker for these cells [16].

“Shock and kill” therapies are based on the idea of inducing productive HIV replication in latently HIV-infected cells; these cells then die due to viral cytopathicity or are removed by the immune system. The “shock and kill” strategy can also be combined with the use of antibody–drug conjugates (immunotoxins) that target and kill cells that express the HIV envelope protein [17]. The neighboring non-infected cells are protected from infection by the ongoing ART [18,19]. This strategy has been repeatedly explored in clinical trials with a number of repurposed drugs [20,21]. Some of them have been shown to activate the hibernating HIV, albeit with little, if any, effect on the size of the latent reservoir [22]. There is a need for a pre-clinical in vivo model to test the wide range of possible experimental strategies.

Prophylactic or therapeutic vaccination: Since the beginning of the HIV pandemic, the HIV research community has devoted enormous effort to finding a prophylactic vaccine against HIV; these efforts have so far been in vain [23]. The major obstacles to finding a vaccine are the high mutation and recombination rates of HIV, which result in immune-escape variants. The cellular and humoral immune responses in HIV-infected individuals are certainly key elements in constraining HIV replication after acute HIV infection; however, they are deficient due to immune-escape variants. Co-evolution of the HIV envelope and antibody responses drives the emergence of bNAbs in a minority of patients. We know today that a combination of two or more bNAbs is able to suppress HIV replication in HIV-infected individuals [24]. However, the major question is the following: can we elicit bNAbs using immunogens? In the current paradigm, the priming stage is critical to the eventual induction of bNAbs. If the appropriate B-cell precursors with potential to develop into bNAbs are not stimulated at this stage, the rest of the sequential vaccine will likely fail [25,26]. In fact, in December 2022, a randomized, double-blind, placebo-controlled phase I study presented the first clinical proof-of-concept results for a germline-targeting vaccine primer [25]. Similarly, huge effort has been expended in the past few decades to develop therapeutic vaccines to enhance HIV-specific T-cell responses [27]. Some approaches have shown promising results in terms of increasing T-cell responses but ultimately have not impacted the overall goal of cART-free remission. DC-based vaccines seem to show the most promise. However, we are still a long way from a therapeutic vaccine.

Pathogenesis: The therapeutic goals listed above are inextricably linked to HIV pathogenesis. Its detailed understanding will open new avenues for HIV cure studies.

## 3. What Models Are Available for Biomedical HIV Research?

The value of humanized mice must be assessed in light of the alternative models for studying HIV and the question(s) being asked. The alternatives are cell lines, primary cells, and non-human primates. Each model type certainly has its place. Cell lines are optimal for studying molecular virology—by virtue of the artificial nature of cell lines, findings should then be verified in primary cells or in an in vivo setting. Primary cells have a finite lifespan and propensity to differentiate; this fact is best illustrated by the differentiation of monocytes to macrophages when adhering to the plastic of cell culture dishes. Primary cells also lack the architecture of the lymphoid organs and all associated aspects, such as cell–cell interactions, cytokine gradients, cellular diversity, etc. Notably, the readily accessible blood cells are less able to elicit ex vivo innate immune responses than cells derived from lymphoid tissue [28], pointing to the need to examine different primary cell types to obtain robust data. Furthermore, the convenience of working with cell cultures in the ambient atmosphere leads to cells being exposed to higher O_2_ levels than they would normally experience in vivo. Oxygen significantly affects the phenotypes of cultured cells and their transcriptional activity [29].

Chimpanzees are permissive to HIV and SIV, while other non-human primates are only permissive to SIV [30,31]. All non-human primate (NHP) models have substantial scientific value in the development of anti-HIV strategies [32]. In particular, the differential susceptibility of sooty mangabeys and rhesus macaques to SIV infection provided a unique opportunity to investigate SIV pathogenesis [2] and permitted researchers to make “dogma-changing” discoveries [2]. Notably, while SIV is closely related to HIV, they differ in various aspects [30,31]. In fact, SIV is more closely related to HIV-2 than HIV-1 [32,33]; thus, insights gained from SIV-infected NHP experiments are very welcome but should be checked for validity for HIV if possible. The main limitations to the use of NHPs are the ethical and cost aspects, which are also reflected in the rather small number of animals used in each experiment. Notably, only a few laboratories in the world have an active research program using non-human primates.

In 2004, the Manz laboratory described the generation of a human adaptive immune system in cord blood cell-transplanted mice [34]. In this seminal work, they showed that the intrahepatic injection of CD34+ human cord blood cells into newborn *Rag2−/−γ_c_−/−* mice that were conditioned by irradiation, leads to the de novo development of B, T, and dendritic cells. This process also led to the formation of structured primary and secondary lymphoid organs and the production of functional immune responses, i.e., human anti-tetanus toxoid (TT) IgG antibodies following TT vaccinations and EBV-specific CD8+ T cells following EBV infection. Our group was the first to demonstrate high-titer disseminated HIV infection in these hu mice [35]. Briefly, we observed higher virulence of CXCR4-tropic strains as opposed to CCR5-tropic strains, as well as the preferential infection of thymocytes by CXCR4-tropic strains. These HIV-infected hu mice failed to generate a robust adaptive immune response. Building on the work by Manz’s group, other laboratories have since adapted and optimized hu mice for their needs.

Subsequent studies using the same or a slightly modified hu mouse model have shown that HIV-infected humanized mice approximate the cardinal features of events that occur in HIV-infected humans—among others, the HIV-mediated depletion of CD4+ T cells [36,37,38,39], virus diversification [40,41,42], the establishment of a latent reservoir [36,43], and the response to anti-retroviral treatment [36]. The close approximation of HIV events to those observed in HIV-infected humans and the ease of manipulating human lymphoid cells in these models make hu mice very valuable to HIV researchers. Indeed, hu mice have enabled HIV researchers to examine a large number of questions that were previously unfeasible to investigate in an in vivo model. In fact, the HIV research community adopted this novel HIV mouse model type very quickly after the first reports. However, we are now faced with the rampant growth of different hu mouse models without any solid comparison between them.

## 4. What Are the Attributes of a Good HIV Mouse Model?

Ideally, a hu mouse model would have a fully fledged human-derived immune system, i.e., with all types of human immune cells, a lymphoid architecture, and the ability to mount adaptive immune responses. In addition, the mice should live for at least ten months without any symptoms whatsoever. In particular, we must pay attention to GvHD as it occurs in both hu PBL mice [44] and BLT mice [45]. GvHD mainly affects the eyes, skin, gastrointestinal tract, liver, and bone marrow [45,46]. GvHD can severely limit the lifespan of the humanized mice and, thus, the time available to conduct experiments. It can also affect the outcome of the experiment. Hu mice are less prone to GvHD.

**Figure 1:**
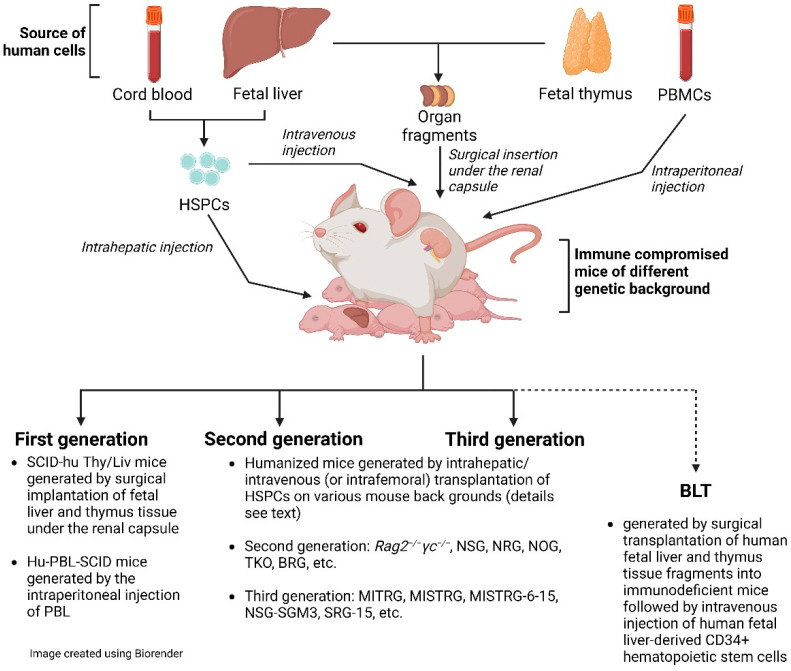
Cartoon of humanized mice.

We can distinguish three generations of hu mouse models (Figure 1). The two most prominent representatives of the first generation are the SCID-hu Thy/Liv mouse model and the hu-PBL-SCID mouse model [6]. Please, note that the SCID-hu Thy/Liv mouse model is not actually a hu mouse model as defined by this definition. Both models have substantial limitations: experiments using the SCID-hu Thy/Liv mouse model are broadly limited to the conjoint organoid, which resembles the human thymus and sustains T-cell lymphopoiesis [47]. Hu-PBL-SCID mice, with significant human blood T-lymphocyte chimerism, suffer from high levels of GvHD and mortality. The time for experimentation is very short due to GvHD [48]. Notwithstanding the limitations of hu-PBL mouse models, they are still used and have value when investigating questions that can be answered in fairly short-term experiments [49,50]. In this review, we focus primarily on a discussion of the value of second- and third-generation hu mice and BLT mice.

The significant improvement in humanization in the second generation of hu mice is due to the very prominent immunosuppression in mice that also lack the common gamma chain (*IL-2Rγ*). IL-2Rγ is required for high-affinity signaling through multiple cytokine receptors, including IL-2, -4, -7, -9, -15, and -21 [51]. Notably, IL-15 is essential for NK cell development [52]. Consequently, IL-2Rγ deficiency eliminates host (murine) NK cells and improves human hematopoietic cell engraftment in immunodeficient recipient mice [4]. There are now a number of mouse strains available with the knock-out of the γ chain (*γ_c_* or shortly *γ*), including non-obese diabetic (NOD/*Scid/IL-2Rγ−/−*)(NSG or NOG), NOD/*Rag1−/−/IL-2Rγ−/−* (NRG), and BALB/c/*Rag2−/−/IL-2Rγ−/−* (DKO/BRG) mice. All these mouse models have a rather high human engraftment level following transplantation with human CD34+ cells. We would like to emphasize that these second-generation hu mice still lack adequate education of human T cells on the mouse MHC molecules in the thymus and provide a suboptimal interaction between human T-cell co-receptors (CD4 and CD8) and mouse MHC [53,54]. In a very recent review by Gillgrass et al. [55], they were considered current-generation models.

We define third-generation mouse models as hu mice that, in addition to immunosuppression, express transgenes of knock-in human molecules, such as HLA, human cytokines, or murine thymic stromal lymphopoietin. In fact, hematopoiesis is a highly regulated and complex process that depends on multiple factors, such as cytokines, chemokines, adhesion factors, etc. [53]. In a human–mouse chimeric model, the interspecies cross-reactivity of key factors might be absent; thus, providing them as transgenes could improve human engraftment both quantitatively and qualitatively. With the exception of mice with human HLA-DR4 and HLA-A2 transgenes [56], which are discussed later, these third-generation hu mice also lack adequate thymic T-cell education for eventual proper T-cell function as second-generation hu mice. Thus, these alterations should result in improvements in humanization—in particular, higher engraftment levels, better functionality of immune cells, and a better lymphoid architecture. Terahara et al. refer to these hu mouse models as “next-generation mouse models” [7].

In addition to the above-described hu mice, which are based on the sole transplantation of CD34+ HSPCs, there is also the bone marrow (B)–liver–thymus (BLT) mouse model [57]. In fact, the BLT mouse model is a further development of the SCID-hu Thy/Liv described by McCune et al. in the 1980s [47,58]. The BLT mouse model is generated by first transplanting fragments of human fetal liver and thymus under the murine renal capsule, followed by irradiation and transplantation with autologous HSPCs. In fact, by providing autologous human fetal thymic fragments as a scaffold, HLA-restricted human T-cell development in vivo is supported. This model is impressive for its high engraftment level and its ability to mount a rather solid adaptive immune response [59]. However, fetal tissue is inherently limited and ethically controversial [60] and, apart from some countries, including the US and China, such fetal tissue is not available by default. BLT mice are created on various mouse backgrounds, including NSG or NRG. Particularly relevant are BLT mice constructed on the background of C57BL/6 *Rag2−/−γc−/−CD47−/−* mice (triple knock-out = TKO) [61]. The additional genetic inactivation of *CD47* negates the requirement for CD47-signal recognition protein α (SIRP-α) signaling and induces tolerance to transplanted human HSPCs. These mice have an intact complement system and show no signs of GvHD up to 29 weeks after transplantation.

Beyond the background mouse strain, there are a large number of variables to consider in humanization, [62] such as (i) the sex of the mice, as female mice show better and faster HSPC engraftment than male mice [63]; (ii) the ultimate HSPCs and their long-term repopulation capacity [64]; (iii) the origin of the HSPCs, i.e., cord blood vs. adult vs. fetal liver; (iv) the expansion of HSPCs [65]; (v) transplantation at newborn or adult age; (vi) the type of injection of HSPCs, e.g., intrahepatic vs. intravenous injection; (vii) the number of HSPCs injected; and (viii) pre-conditioning performed with irradiation or busulfan. Hu mouse models have been customized by different laboratories according to researchers’ preferences, history, and available resources; a recent review by Stripecke et al. proposed minimum requirements for information on the humanized mouse model used when reporting on such studies [62] in order to contextualize the data presented. Beyond the more commonly used HIV mouse models, there are novel mouse models yet to be tested for HIV research, such as mouse models based on the injection of PBMCs into murine MHC class I and II double knock-out NSG [66] or NOG mice [67].

We will now return to the main question: what are the attributes of a good HIV mouse model? We believe that this question must be tackled from a different angle. Indeed, we must first define the research question that we wish to address, define the requirements needed to study the research question, and finally consider whether a particular hu mouse model is appropriate. There is still no universal humanized mouse model for use in the study of HIV infection.

## 5. Of All the Novel Models Reported, Is There a Model That Should Preferably Be Used to Study HIV Infection?

Most laboratories have adapted their humanized mouse models in accordance with their specific goals. We must derive and extrapolate the data from one work to another as related to the advantages and disadvantages of a certain humanized mouse model—direct comparisons of the models are rare and mostly not disease-related. We focus herein on the articles that directly compare different humanized mouse models in the context of HIV. We then discuss which humanized mouse model is suitable to address the questions outlined at the beginning.

The Lishan Su laboratory compared NRG-hu HSC to NRG-hu Thy/HSC mice for the exploration of HIV infection [68]. In this study, we are sticking to the nomenclature provided by the Lishan Su lab—the conventional naming will be hu NRG mice and BLT NRG mice. NRG-hu HSCs were constructed by transplanting fetal-derived HSPCs intrahepatically at newborn age after irradiation. NRG-hu Thy/HSC mice were generated by surgically implanting ~1 mm^3^ fragments of human fetal thymus under the renal capsule in sub-lethally irradiated 5–6-week-old NRG mice, followed 3 h later by intravenous injection of autologous CD34+ cells. The generation of this NRG-hu Thy/HSC mouse model was very close to the original description of the way in which BLT mice are generated. Human cell immune reconstitution was similar, with ~70% in both models as quantified by the number of human cells in the peripheral blood. The authors recognized some “subtle” differences in the cellular composition and time taken to reach the peak HIV viremia level; however, both models were otherwise very similar in terms of cytokine secretion in response to stimulation of the innate immune system, the level of HIV replication and response to cART, the reduction in activation/exhaustion, and in HIV reservoir size after IFN pathway blocking.

Hur et al. investigated in depth the effect of HIV-specific neutralizing IgA on the mucosal transmission of HIV in BLT mice [69]. To deliver anti-HIV IgA in a continual manner, they devised a HSPC-based genetic approach using an *IgA* gene. They found that these antibodies protected from the virus-mediated depletion of CD4+ T cells after vaginal HIV challenge. They repeated the experiments in hu Balb/c *Rag2−/−* mice and obtained the same results. Apparently, the superior human engraftment in BLT mice was not important for this purpose.

In a very recent study by Lepard et al., hu NRG mice were compared with next-generation HLAI/II transgenic hu DRAG-A2 mice (NOD.*Rag1KO.IL-2RγcKO* expressing human HLA-DR4 and HLA-A2; DRAG-A2 mice are also known as DRAGA mice) for their use in HIV research [56]. Very importantly, only cord blood samples that were positive for both the DRB1*04:01 and A*02:01 allele isotypes were used to humanize DRAG-A2 mice. Both hu NRG mice and hu DRAG-A2 mice developed robust human leukocyte reconstitution. Hu DRAG-2 mice showed higher numbers of CD4+ T cells and CD14+ cells. However, HIV infection by vaginal challenge generally resulted in similar HIV replication blood levels and depletion of CD4+ T cells independent of the mouse background.

Holguin et al. compared HIV infection in a hu-PBL model on the background of a new mouse strain, B6.129S-*Rag2^tm1Fwa^ CD47^tm1Fpl^ Il2r^gtm1Wjl/J^*, which lacked *Rag1*, *IL-2Rγ*, and *CD47* (triple knock-out (TKO)), versus hu-PBL NSG [70]. They found that both models supported HIV infection equally well. The TKO mice, however, showed a 24-day delay in the onset of mild GvHD and a 15.5-day delay in the onset of moderate GvHD. Even if GvHD emerges later in TKO mice, it could still confound the results obtained.

In our laboratory, we also investigated HIV replication in hu MISTRG and hu MITRG mice in parallel [71]. MITRG mice are based on a *Rag2−/−IL-2Rγ−/−* background expressing the human M-CSF, IL-3/GM-CSF, and TPO; MISTRG mice have, in addition, a bacterial artificial chromosome (BAC) transgene encoding human *SIRP-α* [72]. Notably, HIV RNA levels were significantly lower in hu MITRG mice than in hu MISTRG mice, with the only difference between these mouse strains being the human SIRP-α transgene [72]. An intact SIRP-α–CD47 axis is key for “do-not-eat me” signaling [73]. Therefore, it stands to reason that phagocytes were involved in the differential dynamics of HIV replication observed, particularly as the clodronate-mediated depletion of macrophages in MITRG mice led to a dramatic increase in HIV replication.

In conclusion, we have only very limited head-to-head comparisons of different humanized mouse models for the study of HIV. Higher immune cell reconstitution per se does not imply a superior HIV mouse model and no clear conclusion can be drawn as to the best humanized mouse model based on the limited number of available comparisons. However, the expression of human genes involved in the immune response can have a major impact on the HIV replication rate and, thus, HIV pathogenesis—hence, different results reported for the same biological question may simply arise from the use of a different humanized mouse model.

Are we able to formulate generally valid requirements for a good HIV mouse model? We propose that a good HIV mouse model should have the following properties: (i) immune cell reconstitution with all types of cells, with an emphasis on HIV target cells; (ii) successful HIV infection in almost 100% of challenged mice; and (iii) be robust enough to endure 12 weeks of experiments. The latter time estimate is a proxy taking into account HIV infection and the time for HIV dissemination, followed by anti-HIV treatment for at least six weeks. The particular requirements, however, may differ according to the research question outlined.

### 5.1. Ad: Which Humanized Mouse Models Are Suitable for Testing Novel anti-HIV Compounds and anti-HIV bNAbs?

Humanized mice, i.e., hu mice and BLT mice, are now a widely accepted pre-clinical, in vivo, small-animal model for the testing new anti-HIV drugs. Indeed, new anti-retroviral drugs or galenic formulations have been investigated in humanized mice. For example, more than a decade ago, we tested the long-acting drug rilpivirin in HIV-infected hu NOG/*SCID/IL-2Rγc−/−* [36]. This work effectively showed: (i) the need for/usefulness of the definition of the pharmacokinetic parameters of investigational drugs in hu mice; (ii) the ability to suppress HIV RNA to undetectable levels with cART; and (iii) the emergence of drug-resistant HIV strains in the case of insufficient dosing. The humanized mouse model used is of secondary importance. Indeed, compound testing has been convincingly performed in hu NSG, NOG, and BLT mice [74,75,76,77,78].

There is also renewed interest in hu-PBL mouse models for the exploration of new anti-HIV compounds [79,80]. However, as stated above, the original hu-PBL mouse model on the SCID background, based on the injection of PBLs, suffers from severe GvHD in cases of high chimerism [81], limiting the experimental time. As an alternative, hu-PBL mice on the background of TKO mice (see above) appear to be of some advantage in this regard. Furthermore, against the backgrounds of NOG and NSG, new mouse models without MHC class I and II have been created [66,67]. Both models, after the injection of PBL, essentially lack GvHD and enable the long-term assessment of human immune responses. These models have yet to be utilized for HIV research; however, they could be particularly useful when larger cohorts of mice are needed. Since HSPCs are cost-limiting resources and humanization via CD34+ cell transplantation is much more time-consuming, it is possible that these hu-PBL models could be of importance in HIV research. Most importantly, anti-retroviral drug concentrations may differ substantially between humans and mice [82,83]; thus, a stringent pharmacokinetic analysis is required prior to testing a drug’s anti-HIV efficacy.

Humanized mice have been key to advances in HIV antibody research [84]. In a landmark study from 2012, a combination of bNAbs was shown to be highly effective at suppressing HIV replication in hu NRG mice [42]. Building upon this initial study, vector-mediated transgenic expression of bNAbs (vectored immunoprophylaxis) [85], additional bNAbs [86,87], bi-specific anti-HIV antibodies [88], combinations of ART and bNAbs [85], and the simultaneous vs. sequential administration of bNAbs [89] have been successfully explored in NRG mice. In HIV-infected hu NRG mice, it has also been shown that the mechanism(s) by which bNAbs suppress active infection is dependent on their ability to bind Fc receptors [90]. Other landmark studies by the David Baltimore laboratory showed the efficacy of the dimeric 2G12 antibody delivered via so-called hybridoma “backpack” tumors [91] using hu *Rag2−/−γc−/−* mice [92], as well as vectored immunoprophylaxis against HIV using hu-PBL NSG mice [93]. In addition, hu NSG mice [94] and BLT mice [95] have also been successfully used to explore the potency of bNAbs.

In terms of testing antibody-mediated Fc receptor function, SRG-15 mice might represent a better humanized mouse model. The SRG-15 mouse is based on the knock-in of human *IL-15* and human *SIRP-α* on a *Rag2−/−IL-2Rγ−/−* background [96]. The transplantation of HSPCs into SRG-15 mice dramatically enhanced the development and functional maturation of circulating and tissue-resident human NK, which may be key to assessing Fc receptor (FcR) dependence. Incidentally, the quantity and quality of CD8+ T cells were also increased in these hu mice. Rajashekar et al. injected these SRG-15 mice with human PBL and demonstrated their significant HIV susceptibility [97] Using this hu PBL SRG-15 mouse model, they showed that a CD4 mimetic along with anti-HIV antibodies resulted in a decrease in HIV replication, the HIV reservoir size, and viral rebound after cART interruption. Furthermore, they convincingly demonstrated that the observed anti-HIV effects were mediated by NK cells; the effect was lost in hu PBL SRG-15 mice lacking human NK cells. As outlined above, the anti-HIV activity of the bNAbs and their dependence on the Fc receptor has been previously demonstrated in hu NRG mice, which may be related to the presence of murine and human macrophages. However, we lack a direct comparison between different mouse strains and must rely on inferred reasons when arguing that one model is superior to another. If Fc-receptor-mediated activity is considered to be of importance to the research goal, it might be preferable to choose a humanized mouse model that supports NK cell function via the transgenic expression of IL-15.

### 5.2. Ad: Development of Cell and Gene Therapy to Cure HIV

Humanized mice have been incredibly valuable in exploring the feasibility of gene therapies, i.e., for exploring the effects of genetically manipulated HSPCs or immune cells on HIV infection. In a seminal study, Holt et al. demonstrated the successful modification of HSPCs by zinc-finger nucleases designed to disrupt CCR5 in hu NSG mice [98]; these humanized mice showed lower HIV levels and preserved human cells throughout their tissues. Since then, different humanized mouse models have been used to study the various targets affecting HIV infection or different gene-targeting techniques and siRNA/microRNA approaches. To a great extent, NSG mice transplanted with genetically modified HSPCs [99,100,101,102,103] or infused with genetically modified T cells [102,104,105] have been used. NSG mice transplanted with genetically modified T-cells are hu PBL-like mice with all the limitations discussed above. Alternatively, BLT mice have been used [106,107,108,109]. It is most likely of little importance which humanized mouse model is used when targeting HIV components, such as the LTR [110]. The same applies for CCR5 as this cell surface marker is present on the corresponding human immune cells in every humanized mouse model. We also believe that when studying the effectiveness of CAR T-cell therapy, it is not important which humanized mouse model is used; in any case, there is a lack of evidence to recommend a specific humanized mouse model. The major challenge will be to find new means of gene delivery that do not require ex vivo gene transduction, myeloablation, and transplantation [111]. Humanized mice will be a very valuable tool in this work [112].

### 5.3. Ad: Exploring Experimental Strategies to Eradicate Dormant HIV

Shortly after the first description of humanized mice serving as a new HIV mouse model, a number of reports documented HIV latency in various models, inter alia, in cART-treated HIV-infected hu *Rag2−/−γc−/−* mice, BLT mice, and hu NOG mice [36] via ex vivo reactivation assays or viral rebound following the analytical interruption of cART. In a seminal study, a mixture of bNAbs in concert with three latency-reversing agents, i.e., an HDAC inhibitor, a BET protein inhibitor, and a T-cell inhibitory pathway blocker, succeeded in curing HIV in the majority of hu NRG mice [90]. Another very intriguing approach, which is known as the “selective elimination of host cells capable of producing HIV” (SECH), was described using hu NSG-SGM3 mice and achieved the permanent clearance of HIV in >50% of mice [113]. SECH is based on administering compounds to trigger viral reactivation and compounds to induce apoptosis and block autophagy. The idea behind SECH is that the apoptosis-inducing proteins take over exclusively in HIV-infected cells [113]. When SECH was applied subsequently to effective cART, complete clearance of HIV infection was observed. Notably, the triple transgenic NSG-SGM3 (NSGS) mice express the human IL-3, GM-CSF, and SCF (013062-NSG-SGM3 Strain Details (jax.org, accessed on 17 January 2023)).

Most studies evaluating latency-reversing agents have used BLT mice [114,115,116,117]. Other hu mouse models will most likely be equally effective for testing the efficacy of latency-reversing agents, such as hu NSG-SGM3 mice treated with SECH. The main challenge will be the systemic clearance of HIV-infected cells that were kicked (shocked) out of latency in humanized mice. In particular, as discussed in more detail below, BLT mice have a superior adaptive immune response to hu mice. Therefore, if the scientific goal is to assess the systemic clearance of HIV-infected cells that were kicked out of latency by the immune system, we would use BLT mice. [118] However, BLT mice have major drawbacks: they start to develop GvHD coincident with human reconstitution and, 22 weeks post-transplantation, they die [45]. Therefore, time will be a limiting factor when studying HIV latency in BLT mice. Furthermore, it will remain questionable if the immune response in BLT mice is solid enough for this systemic clearance. One advancement is the aforementioned GVHD-resistant C57BL/6 *Rag2−/−γc−/−CD47−/−* triple knock-out (TKO)-BLT mouse [61]. This mouse model has an additional 15–18 weeks of healthy longevity compared with other BLT models and, thus, provides substantially more experimentation time [119].

Meanwhile, a number of studies have succeeded in defining the cell subsets that preferentially harbor latent HIV [16]. Hu NSG mice very satisfactorily showed the same enrichment of latent HIV in PD-1+ and TIGIT+ CD4 T cells [120]. Thus, hu mice mirror the cardinal features of HIV latency observed in HIV-infected humans.

A recent study exploring a novel assay to quantify latency reversal and viral clearance achieved this result using the antibody-mediated killing of HIV-infected cells in hu MISTRG-6-15 mice [121]. Hu MISTRG-6-15 mice were created by knocking in MISTRG mice with human IL-6 and IL-15 [122]. The lab that created this hu MISTRG-6-15 mouse model showed higher numbers of circulating NK cells and monocytes, as well as a higher number of NK cells in the tissues. They also had higher cytolytic activity than NK cells from hu NSG mice. Since no direct comparison was performed, it remains unclear whether this model is superior to more recent hu MISTRG or SRG-15 mice.

The Harris Goldstein Laboratory recently reported the establishment of a novel model to investigate the in vivo activation and depletion of a patient-derived latent HIV reservoir [123]. This model is based on the injection of PBMCs into the spleens of NSG mice from an HIV-infected individual under cART with an undetectable viral load, along with irradiated PBMCs from a healthy volunteer. Using this model, the Goldstein lab demonstrated superior activity in delaying the viral rebound with a bNAb with preserved Fc-mediated effector responses compared to a bNAb lacking Fc-mediated effector responses.

With this brief overview of the different humanized mouse models to study HIV latency, we would like to reiterate the importance of defining which features are needed in the model chosen. In general, humanized mice, irrespective of their mouse background and customization, have proven to be valuable platforms for the investigation of HIV latency.

### 5.4. Ad: Prophylactic or Therapeutic Vaccination

Vaccination studies require a solidly functioning immune system, i.e., the ability to generate antibodies with breadth and magnitude, as well as a specific T-cell response as in humans. We reported in our pioneering study that only 1/25 HIV-infected hu *Rag2−/−γc−/−* mice mounted a detectable HIV-specific IgG response [35]. Our data on the lack of a solid humoral immune response were corroborated in HIV-infected NOG mice [124]. Contrarily to the humoral immune response, hu NSG mice apparently generate an HIV-specific cellular immune response [125]. Indeed, Gorantla et al. showed the presence of HIV-specific CD8+ T cells through their ex vivo incubation with overlapping HIV env and gag pools. Furthermore, they noted also a significant increase in HIV load following the depletion of CD8+ T cells either 2 or 5–7 weeks after HIV infection.

The partial failure to mount a solid immune response was attributed to the lack of HLA expression as a critical element needed to support the homing and education of human pro-T cells in the mouse thymus [126]. The introduction of HLAI/II genes into NRG mice should correct this issue. Indeed, hu NRG mice expressing HLA-DR4 molecules (DRAG mice) showed solid immunoglobulin class switching, while hu NRG mice expressing HLA-A2 molecules—as well as hu NRG mice expressing HLA-A2 and HLA-DR (hu DRAG-A2 or DRAGA)—generated significant numbers of functional cytotoxic CD8+ T cells against the influenza GIL peptide [126]. More recently, data on the immune function potential of hu DRAG-A2 mice in HIV infection were reported [127]. Ollerton et al. found modest levels of HIV gp41-specific human IgM and IgG in HIV-infected animals at each time point during a follow-up period of 42 to 133 days post-infection. They attributed the sub-optimal antibody development to the impairment of the germinal center reaction. The characterization of the HIV-specific antibodies was lacking. Notably, this group extensively analyzed the secondary lymphoid tissue in these HIV-infected hu mice, for which they must be congratulated. Thus, although human HLAI/II molecules were present, hu mice apparently do not mount an efficient adaptive immune response against HIV.

In contrast to the weak or inconsistent humoral immune response in HIV-infected hu mice, it is possible to induce a decent humoral immune response in hu NRG mice via a CD40-targeting vaccination strategy [128]. In fact, administering a construct consisting of anti-CD40 antibody fused to the HIV envelope gp140 protein with either a TLR9 agonist (CpG) or NYVAC-KC vector (NYVAC-KC vector: replication-competent, attenuated recombinant of the vaccinia virus strain, NYVAC) encoding the homologous Env gp120 using a prime/boost scheme resulted in Env-specific IgG-switched human memory B cells. Furthermore, both vaccination strategies induced splenic germinal center-like structures containing human B and human T follicular helper (Tfh)-like cells. It would be interesting to observe whether this vaccination strategy could protect against an HIV challenge.

Hu third-generation models may be superior in their ability to mount an adaptive immune response against HIV. As far as we are aware, no studies have reported superior adaptive immune responses, including the HIV-infected hu DRAG-A2 mice, as discussed above. Hu MISTRG mice excel in terms of their myeloid compartment but, apparently, their humoral immune response is low and they are similar to hu NSG mice in response to ovalbumin immunization [72]. A very interesting new hu mouse model was reported wherein lymph node development was restored by the transgenic expression of murine thymic-stromal-cell-derived lymphopoietin in Balb/c *Rag2−/−IL2Rγc−/−Sirp-α*^NOD^ (BRGS) mice [129]. These hu mice showed a larger thymus, more mature B cells, and abundant IL-21 Tfh cells; they also showed enhanced antigen-specific responses. These mice were very susceptible to HIV infection but did not show any anti-HIV specific antibodies, which the authors ascribed to the strong virus-induced depletion of immune cells. Moreover, an improvement in lymph node organogenesis was obtained via the selective expression of the γc gene in the lymph tissue inducer lineage by using the endogenous promoter of RORγ t [130]. To further increase the likelihood of a good humoral immune response, these mice were back-crossed with GM-CSF/IL-3 transgenic NOG mice (GM3 mice). Indeed, the humanization of NOG-*pRORγt-γc/GM3* mice resulted in a more robust OVA-specific human IgG titer. Investigation of HIV infection and its immune response in this model is pending.

By far the most convincing humanized mouse model for the study of HIV-specific adaptive immune responses is BLT mice. HIV in BLT mice results in positive HIV Western blots and HIV-specific T-cell responses [59]. BLT mice infected with HIV show the rapid evolution of HIV to functional CD8+ T cells, similarly to HIV-infected humans [131]. BLT mice with exhausted immune responses (i.e., high expression of PD-1 on CD8+ T cells) in the context of HIV infection respond favorably to PD-1 blockade, with transient decreases in HIV load [132]. A CD40-targeting vaccination strategy in concert with a TLR3 agonist was investigated by Lishan Su’s laboratory in their BLT NRG mouse model [133]. They found a solid anti-HIV cellular response in uninfected BLT NRG mice. In HIV-infected BLT NRG mice under effective cART in a therapeutic setting, this vaccination induced HIV-specific CD8+ T cells and reduced the level of the HIV reservoir. Furthermore, this vaccination strategy resulted in a delay in viral rebound subsequent to cART interruption. However, not all papers have been so enthusiastic about the ability of BLT mice to mount a humoral immune response. Biswas et al. immunized BLT mice repeatedly with an adjuvanted HIV gp140 [134] that did not trigger class switching; the levels of specific IgM remained unchanged. In addition, they found that the B5+ B-cell subset may play a role in the vaccine-induced antibody response. Similarly, a recent paper pointed to a less than optimal reconstitution of innate immune cells in BLT mice, which blunts the T-cell responses to immune challenges [135].

To conclude, humanized mouse models still need optimization for the straightforward study of adaptive immune responses. Based on current knowledge, BLT mice would be the preferred model for this purpose. It would be highly interesting to explore whether the construction of a BLT mouse model in the background of an MISTRG/MISTRG-6-15 or 3SGM mouse would result in the generation of a complete functional human immune system [119,136].

As outlined above, researchers in the vaccine field are investigating germline-targeting vaccine priming. In fact, such an approach has yet to be tested in humanized mice to determine whether it offers any meaningful result. Considering that the induction of bNAbs requires the priming of precursor B cells, selection of B cells, affinity maturation, and all cellular interactions within the sophisticated lymphoid architecture, we doubt that the humanized mice available today have any value in this regard.

Therapeutic vaccination is currently in vogue, and, in contrast to preventive vaccination, aims to strengthen/induce an efficient immune response in patients who are already infected. The purpose of therapeutic vaccination is to modify the host’s immune response to HIV, either by redirecting it to the most conserved/vulnerable regions of the HIV viral proteome to mimic the response of spontaneous controllers [137] or by increasing its depth to cover more viral diversity [138,139]. The ultimate goal would be to control HIV replication without the need for ongoing cART. Therapeutic vaccination requires an immune system that is initially responsive and largely intact in order to test different booster strategies by whatever means [140]. As elaborated above, the currently available humanized mouse models do not present such a functional immune system and, in our opinion, are not a reliable HIV mouse model for the evaluation of therapeutic vaccines for HIV. However, as a word of caution, the laboratory of Lishan Su has generated some promising data as related to therapeutic vaccination in their BLT NRG model, as discussed above [133].

### 5.5. Ad: HIV Infection via the Vaginal Route

Women and girls accounted for 49% of all new HIV infections in 2021 (Fact Sheet, UNAIDS). This points to the great importance of preventing HIV infection via the vaginal route. There are numerous papers demonstrating the efficacy of some types of drugs to prevent HIV infection via the vaginal route in BLT mice and hu mice. However, might the events in mice approximate the events occurring in human HIV transmission via the vaginal route?

Deruaz and Luster very succinctly summarized the anatomy of the mouse female genital tract and concluded that it is very similar to the human one, despite its smaller size [141]. Therefore, humanization in mice should generate a pre-clinically relevant model for the study of HIV in the female reproductive tract. Indeed, the BLT mouse model shows a satisfactory reconstitution of the female reproductive tract [142]. It has proven useful, inter alia, for prevention studies investigating the efficacy of 1% tenofovir [143] or CD4 aptamer-siRNA chimeras [144] applied topically, or of antiretroviral drugs [142,145,146] or bNAbs [147] administered systemically. A very intriguing study showed superior efficacy of IgG1 compared to IgG2 in preventing vaginal HIV transmission when administered via vectored immuno-prophylaxis [148]. The authors of the latter study explained the reduced protective effect of IgG2 by the lack of Fc-mediated functionality. The superior protective effect of IgG1 is most likely due to monocytes/macrophages as NK cells are rather rare in the BLT mouse model [148]. The BLT mouse model was also very valuable for assessing the localization of HIV target cells and their shedding into cervico-vaginal secretions [149]. Following HIV infection, there were increased numbers of CD4+ and CD8+ T cells and HIV was present in cervico-vaginal secretions. cART efficiently suppressed HIV in cervico-vaginal secretions and restored CD4+ and CD8+ T-cell numbers.

What can be said of the reconstitution of the female reproductive tract in humanized mice? The first studies were controversial regarding the extent of mucosal tissue reconstitution [145,150]. A recent study by Nguyen et al. explored which factors determine successful HIV transmission by the vaginal route. Using hu NRG mice, they found that the frequency of human CD45+ target cells in peripheral blood was the main criterion for intravaginal HIV infection, followed by the viral dose [151]. Notably, hu NRG mice with >10% human reconstitution had a significantly greater proportion of human CD4+ T cells in the vaginal mucosa than hu NRG mice with <10%. Similarly, Stoddart et al. reported higher susceptibility to vaginal HIV transmission in BLT-NSG mice compared to BLT-NOD-SCID mice, with the former showing substantially higher reconstitution with human cells [152]. Therefore, the initial controversy regarding which humanized mouse model is the best for vaginal studies is most likely explained by the different extents of human immune reconstitution, which was less satisfactory in the initial mouse models reconstituted with human cord-blood-derived HSPCs [150]. Over the past decade, humanized mice have been used successfully, inter alia, to study the protective effect of both intravaginally applied bNAbs [153] and the polymeric IgA form of anti-HIV antibodies given systemically, which results in protection against virus-mediated CD4+ T-cell depletion [69]. Hu NRG mice have also been used to study the effects of depot medroxyprogesterone acetate (DMPA) on vaginal HIV transmissibility [154]. DMPA is a widely used hormonal contraceptive in sub-Saharan Africa. DMPA resulted in enhanced FITC–dextran dye leakage from the vaginal tract into the systemic circulation, enhanced target cells in the vaginal tract, and increased the rate of intravaginal HIV infection. Notably, Veselinovic et al. effectively summarized the methodology by which HIV-1 mucosal transmission and prevention can be modeled in hu mice [155].

HIV challenges via the rectal route have also been successfully examined in BLT mice and in hu mice. However, the characterization of the human immune system in the gastrointestinal tract is much less studied than that in the female reproductive tract. Notably, the systemic administration of antiretroviral drugs prior to exposure prevented rectal HIV transmission in BLT mice [156]. In hu NRG mice, non-neutralizing antibodies targeting the HIV envelope did not induce sterilizing immunity when mice were rectally challenged with HIV; rather, it reduced the viral load burden [157].

In conclusion, hu mice and BLT mice are useful small-animal models for the study of HIV pathogenesis, treatment, and prevention in the female reproductive tract. The key factor required for successful work in this area is a high human engraftment rate. In this regard, we would point to a recently published paper describing the superior engraftment of human immune cells in the gastrointestinal tract in humanized NRG mice [158]. The researchers achieved this superior engraftment by first transplanting human cord blood-derived CD34+ cells at newborn age, followed by the intraperitoneal injection of donor-matched umbilical cord blood cells at 12 weeks of age in concert with up to four injections of IL-7.

In summary, the major variables for vaginal challenge studies are: (i) the way in which HIV is applied vaginally; (ii) the infectious dose; (iii) whether progestin has been applied to thin the vaginal layer; and (iv) the level of human engraftment. The question also remains open as to whether whole-body irradiation leads to ovarian insufficiency or direct irradiation damage to the female reproductive tract. As far as we know, no data exist regarding this topic. Regarding vaginal HIV studies, it should be noted that, for experimental reasons, we aim for a 100% HIV transmission rate, which is quite the opposite to what is observed in humans, with a vaginal transmission rate below 1% [159]. This huge discrepancy opens up a great deal of scope for speculative extrapolations from data gathered in humanized mice to humans.

### 5.6. Ad: Pathogenesis

This research area is huge and diverse; we selected a limited number of published works for inclusion in this review. A cardinal feature of HIV infection is its diversity due to the failure-prone reverse transcriptase and recombination rate [160]. Indeed, using hu *Rag2−/−γc−/−* mice, the mean rate of divergence of viral populations in mice was similar to that observed in a cohort of humans during a similar period of infection [40]. A technical note presented single-cell RNA seq technology to study HIV-induced transcriptomic changes in innate immune cells in lymphoid organs [161]. The researchers used HIV-infected hu NRG mice and found that TRAIL was upregulated in the innate immune cells; its blockage prevented HIV-induced CD4+ T-cell depletion without affecting the HIV load. Another study used TKO-BLT mice to show that blocking CCL2 reduced the seeding of the latent HIV reservoir following repeated HIV challenges via the rectal route [28]. In contrast to CCL2’s pro-HIV effect, IL-21, by inducing microRNA-29 in CD4+ T cells, resulted in a lower number of HIV-infected BLT mice two weeks after the IV injection of HIV [162]. Finally, a recent article attracted our attention. In this article, metformin targeting the mitochondrial respiratory chain complex suppressed HIV replication in hu NRG mice [163]. In this work, the authors used CD4+ T cells and patient samples as well as the aforementioned hu mice; all aspects fitted well together, confirming the value of an integrated approach and, particularly, the value of hu mice in studies of HIV pathogenesis.

As already discussed above, third-generation hu mice may be particularly useful when studying certain cell types, e.g., NK cells, which are somewhat dysfunctional in second-generation hu mouse models. A number of different mice that express human IL-15 [96,122,164,165], some in concert with either IL-6 [122] or IL-7 [165] and on various mouse backgrounds, have been reported. Therefore, these hu mouse models are most likely better suited to research questions involving NK cells; a head-to-head comparison, however, is lacking. The importance of a human transgene for a particular cell type is also well-illustrated by comparing BLT mice ± human IL-34 [166]. The BLT mice with IL-34 show the extensive reconstitution of human microglia in the brain, which supports HIV replication.

Finally, we would like to mention two elegant approaches, by which humanized mice can be used to study HIV pathogenesis, that were presented by the Garcia laboratory [167,168,169]. This research group benefited from diverse mouse strains. In analogy to the early SCID-hu Thy/Liv model [47,58], they implanted NOD/SCID and NSG mice with human fetal liver and thymus tissue under the renal capsule without injection of human CD34+ cells [167]. Humanization in this way resulted in humanized mice with only T cells and no myeloid cells. These so-called T-cell-only mice (TOM) are highly susceptible to HIV and can efficiently maintain virus replication. HIV-infected TOM undergoing ART harbor latently infected resting CD4+ T cells. This TOM model is highly valuable to studying HIV latency provided that the focus lies exclusively on T cells and disregards other cell types. In addition, this group presented the MOM model; MOM stands for macrophage-only mice. MOM are generated by transplanting NOD/SCID mice with human CD34+ cells, which results in the reconstitution of human myeloid cells and B cells; however, they are completely devoid of human T cells [168]. Using these mice, researchers showed that macrophages alone can sustain HIV replication in the absence of T cells [168] and may also serve as an HIV latency reservoir [169].

## 6. Conclusions

Hu mice are a valuable tool for HIV researchers. Although there are limitations to the use of humanized mice, as elaborated in this review, we have obtained great insights into many diverse aspects of HIV by studying them. We must reiterate that humanized mice are merely an in vivo model and the researcher needs to seek the best-suited model to answer the questions they have posed. Furthermore, head-to-head comparisons between humanized mouse models would be extremely helpful in appreciating their individual value.

## Data Availability

Not applicable.

## Abbreviations

AIDS	Acquired immunodeficiency syndrome
ART	Antiretroviral therapy
BLT	Bone marrow thymus liver
bNAbs	Broadly neutralizing antibodies
BRG	*Rag2−/−/IL-2Rγ−/−* in BALB/c background
cART	Combined antiretroviral therapy
CCR5	C-C chemokine receptor 5
COVID-19	Coronavirus disease 19
CXCR4	C-X-C chemokine receptor type 4
DARPins	Designed ankyrin repeat proteins
DMPA	Depot medroxyprogesterone acetate
Env	Envelope protein
FITC	Fluorescein isothiocyanate
GM-CSF	Granulocyte–macrophage colony stimulating factor
GvHD	Graft versus host disease
HIV	Human immunodeficiency virus
HLA	Human leukocyte antigen
HSPCs	Hematopoietic stem and progenitor cells
hu	Humanized
Hu-PBL-SCID	Humanized peripheral blood leukocytes severe combined Immunodeficiency
IgA	Immunoglobulin A
IgG	Immunoglobulin G
IL	Interleukin
MHC	Major histocompatibility complex
MISTRG	*M-CSF^h/h^ IL-3/GM-CSF^h/h^ SIRP-α^h/m^ TPO^h/h^ RAG2−/−IL-2Rγ−/−*
MITRG	*M-CSF^h/h^ IL-3/GM-CSF^h/h^ TPO^h/h^ RAG2−/−IL-2Rγ−/−*
NK cells	Natural killer cells
NOD	Non-obese diabetic
NRG	*Rag1−/−/IL-2Rγ−/−* in NOD background
NSG	*SCID/IL-2Rγ−/−* in NOD background
NSG-SGM3	NSG-*Human Stem cell factor–GM-CSF–IL-3*
NYVAC-KC	Replication-competent, attenuated recombinant of the vaccinia virus strain NYVAC
PBMCs	Peripheral blood mononuclear cells
PD-1	Programmed cell death protein-1
*Rag2−/−γc−/−*	*Rag2/Il2rγ* compound mutant mice
RNA	Ribonucleic acid
SCID-hu Thy/Liv	Severe combined immunodeficiency–human thymus and liver
SIRP-α	Signal recognition protein alpha
SIV	Simian immunodeficiency virus
SRG-15	Human *SIRP-α* and human *IL-15* knock-in
TKO	Triple knockout
TLR	Toll-like receptor
UNAIDS	Joined United Nations Programme on HIV/AIDS
